# Bilateral Dentigerous Cyst in Impacted Mandibular Third Molars: A Case Report

**DOI:** 10.7759/cureus.3691

**Published:** 2018-12-05

**Authors:** Haymenth Vasiapphan, Pradeep J Christopher, Srivatsa Kengasubbiah, Vandana Shenoy, Senthil Kumar, Ashwan Paranthaman

**Affiliations:** 1 Oral and Maxillofacial Surgery, Thai Moogambigai Dental College & Hospital, Chennai, IND

**Keywords:** bilateral dentigerous cyst, non-syndromic, unerupted tooth

## Abstract

A dentigerous cyst is an odontogenic cyst, associated with the crown of an impacted or unerupted tooth. The occurrence of bilateral dentigerous cysts is uncommon among the odontogenic cysts of the jaw, as they are usually solitary. Multiple cysts, when reported, are generally associated with syndromes such as cleidocranial dysplasia, Maroteaux-Lamy syndrome, and systemic diseases like mucopolysaccharidosis. This article presents a case of bilateral mandibular dentigerous cysts in a nonsyndromic patient, along with a review of the literature and an examination of the treatment modality.

## Introduction

A dentigerous (follicular) cyst is a developmental odontogenic cyst usually attached to the crown of an unerupted tooth. The epithelial cells from the reduced enamel epithelium form the lining of the cystic lesion. It is the second most common type of odontogenic cyst and is a fluid-filled sac that develops in the jaw bone and adjacent soft tissue. Mandibular third molars, permanent maxillary canines, and mandibular premolars are commonly involved [1]. Dentigerous cysts are usually asymptomatic and found incidentally during the evaluation of an unerupted tooth. The most common signs and symptoms are pain and bone expansion [2]. Histological diagnosis plays a key role in the definitive diagnosis. Complications resulting from a dentigerous cyst are rare but may involve tooth displacement and the maxillary antrum and nasal cavity obliteration. It can also cause paresthesia of the inferior alveolar nerve or metaplastic or dysplastic changes [3]. The proper treatment is enucleation of the cyst and removal of the impacted or unerupted tooth. When complete excision of the cyst is achieved, the prognosis is good and the recurrence rate is low [2].

## Case presentation

**Figure 1:**
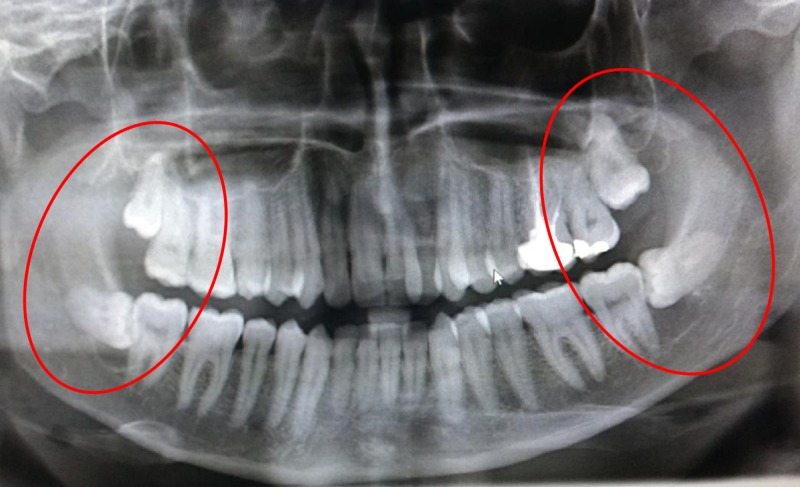
Panoramic radiograph showing radiolucencies compatible with dentigerous cysts, associated with both horizontally impacted mandibular third molars (red circles)

**Figure 2:**
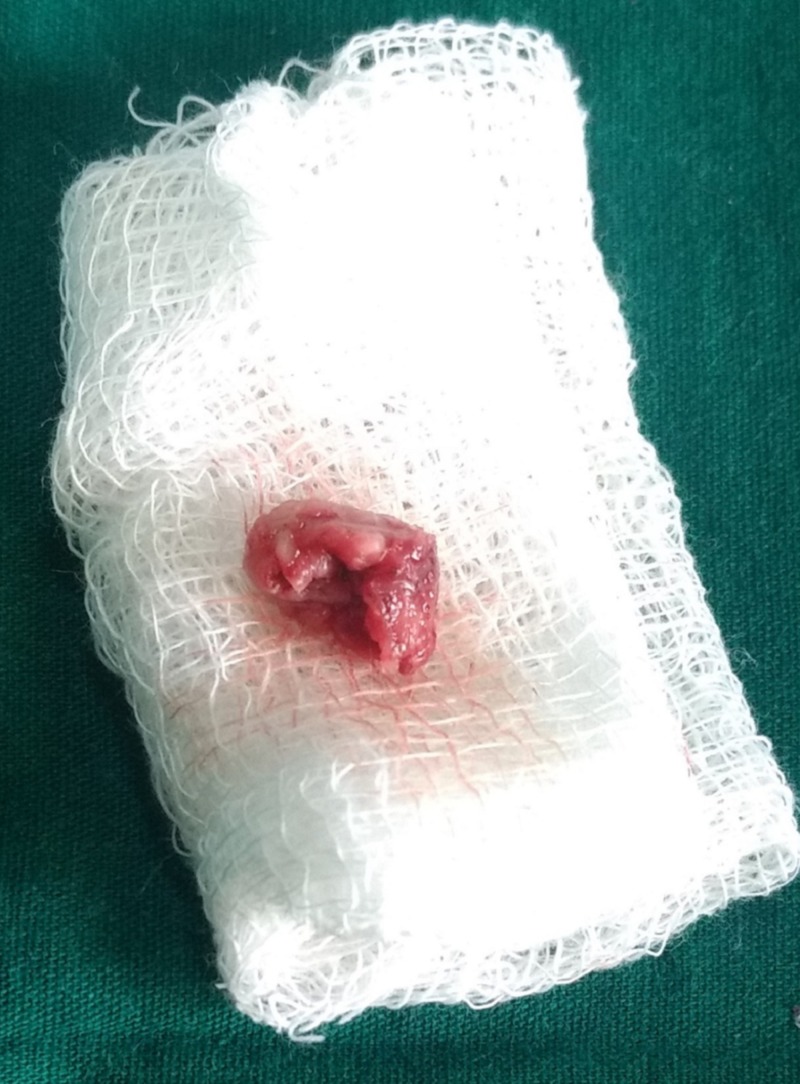
Excised specimen from the right side

**Figure 3:**
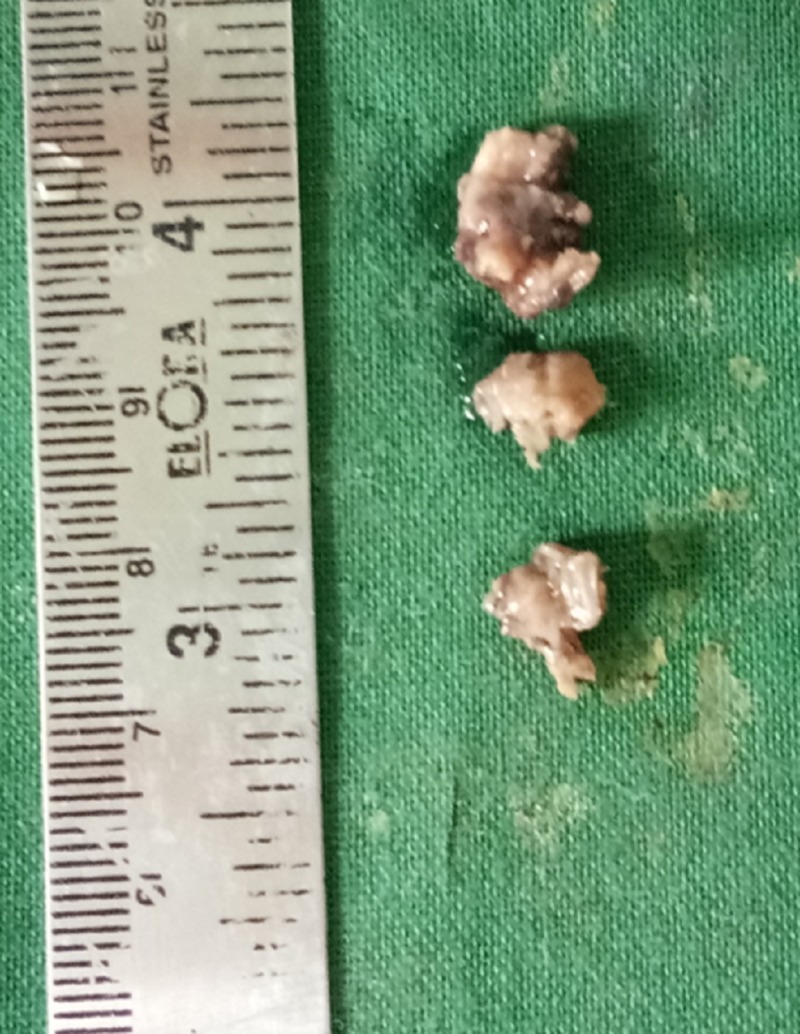
Excised specimen from the left side

**Figure 4:**
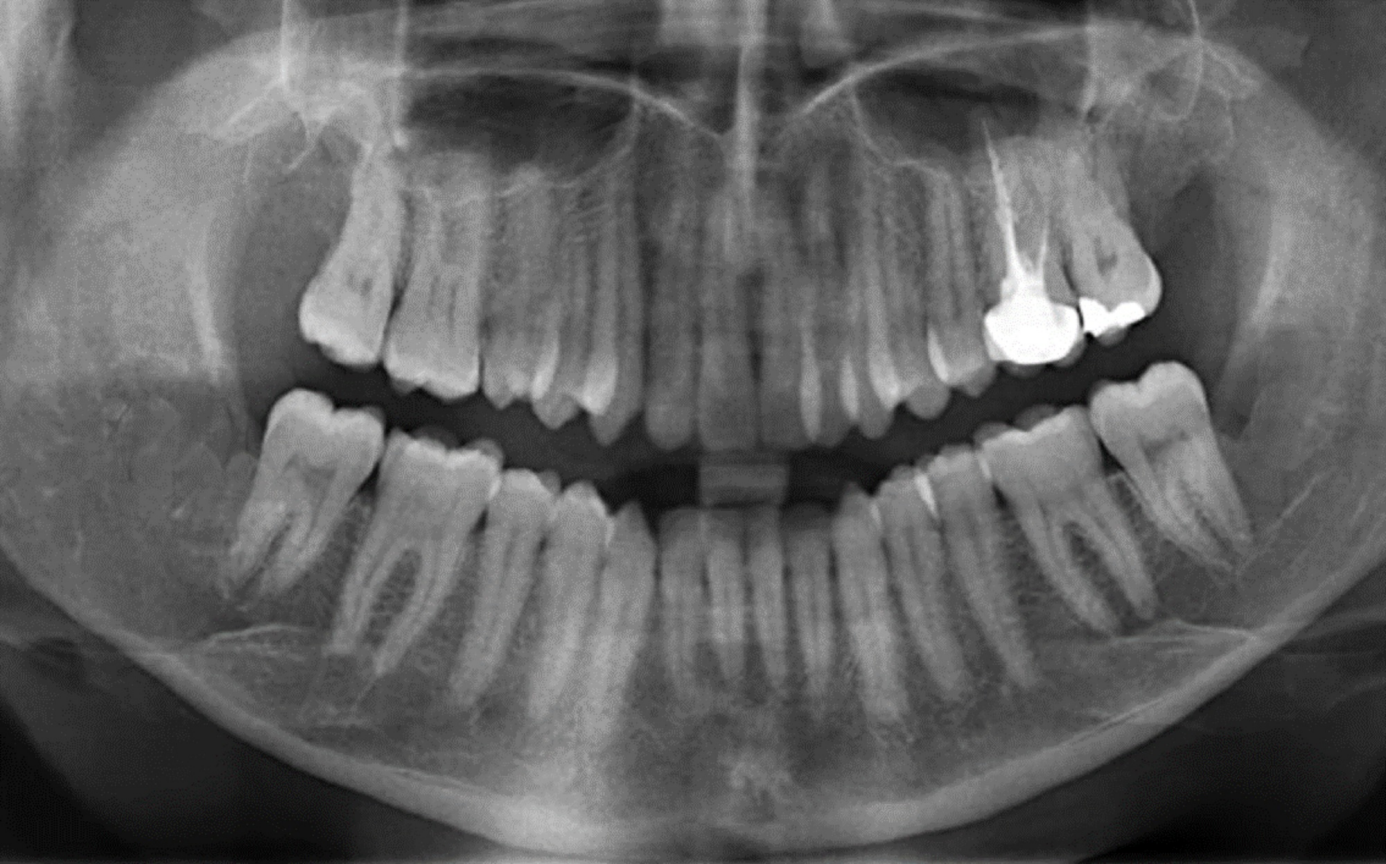
Follow-up panoramic radiograph after six months, showing good bone formation with no recurrence

A 27-year-old male patient reported to the department of oral and maxillofacial surgery at Thai Moogambigai Dental College and Hospital in Chennai, Tamil Nadu, India. The patient’s chief concern was a pain in his left upper back tooth region lasting a week. The pain was dull, aching, continuous, and nonradiating, with no aggravating or relieving factors associated with the left upper back tooth region. The patient is healthy without systemic diseases or deleterious habits. On an intra-oral examination, we noted that all the third molars were clinically unerupted. On careful inspection, we noted his oral cavity appeared to be normal without any buccolingual swelling or mucosal changes. His mouth opening was normal. A dental panoramic radiograph revealed an accidental finding of cystic lesions associated with the lower third molars bilaterally. A review of his orthopantomogram revealed the upper third molar were impacted with a sinus approximation and a well-defined, unicystic radiolucency around the impacted lower third molars bilaterally. The lesions had bilaterally enclosed the crown of the horizontally impacted lower third molars from the cementoenamel junction, and they extended inferiorly to the apex of the distal root of the respective second molars (Figure 1). Surgical excision of the lesion along with the impacted molars was planned under local anesthesia. A modified Ward’s incision was placed, and the mucoperiosteal flap was reflected. Bone guttering was done under copious saline irrigation, and the tooth was sectioned and removed. The soft cystic tissue was exposed, and we performed a complete enucleation of the cysts (Figures 2-3). Carnoy’s solution was applied to cauterize the remnant cystic lining on the cavity wall junctions, and hemostasis was achieved. The obtained specimens were biopsied. Povidone-iodine was flushed into the sockets generously. Wound closure was done with a 3-0 silk suture. The patient was evaluated again three days later and had no paraesthesia. After one week, the sutures were removed and the wound healed satisfactorily. The histopathological report revealed a dentigerous cyst bilaterally. It showed a thin fibrous cystic wall lined by two-to-three cell layers of thick, nonkeratinized, stratified squamous epithelium. The connective tissue showed an inflamed infiltrate. Follow-up examinations at one, three, and six months yielded no degenerative changes around the third molar region, and no pockets formed in the distal aspect to the lower second molars (Figure 4).

## Discussion

Dentigerous cysts usually occur as a solitary cyst and present in patients three to 57 years old but are most commonly seen in the second and third decades of life [3]. Dentigerous cysts are commonly seen in mandibular third molars, maxillary canines, and mandibular premolars, and they rarely involve deciduous teeth. The bilateral presence of this cyst is very rare and is associated with Maroteaux-Lamy syndrome, mucopolysaccharidosis, cleidocranial dysplasia, and the long-term consumption of cyclosporine and calcium channel blockers [4]. The cystic cavity is lined with the epithelial cells derived from the reduced enamel epithelium of the tooth-forming organ. Pathogenesis of the cyst may be due to the pressure exerted by an erupting tooth that obstructs venous flow, inducing the accumulation of exudates between the reduced enamel epithelium and the tooth crown [5]. Dentigerous cysts usually arise from the follicular epithelium, and they have an increased potential for growth, differentiation, and degeneration. These cysts are lined by nonkeratinized stratified squamous epithelium. Radiographically, dentigerous cysts are suspected when the size of the follicular space is larger than 5 mm. The lesion appears to be radiolucent and unilocular with well-defined sclerotic borders and is associated with the crown of an unerupted tooth. Dentigerous cysts can cause the displacement of the adjacent tooth and the resorption of the roots [2]. The differential diagnosis may include odontogenic keratocysts, primordial cysts, and odontogenic tumors (e.g., Pindborg tumor, adenomatoid odontogenic tumor, mural ameloblastoma, unilocular ameloblastoma, ameloblastic fibroma, odontomas, and cementomas) [1]. The most common clinical complication is paresthesia of the inferior alveolar nerve.

The treatment of dentigerous cysts is enucleation of the cysts and removal of the impacted or unerupted tooth. Large cysts like these can be marsupialized initially to decompress the cystic contents and enucleated more conservatively. Marsupialization is advised as a treatment modality for dentigerous cysts in children [2]. The main drawback of marsupialization is that the pathological tissue remains in situ (Table 1).

**Table 1:** Literature review of bilateral third molar dentigerous cysts not related to syndromes

Author	Age/sex	Year	Treatment
Myers et al. [6]^a^	19/Female	1943	Enucleation
Callaghan et al. [7]	38/Male	1973	Enucleation
Burton et al. [8]	57/Female	1980	Enucleation
Crinzi et al. [9]	15/Female	1982	Enucleation
Banderas et al. [10]	38/Male	1996	Enucleation
Ko et al. [6]	42/Male	1999	Enucleation
Shah et al. [11]	39/Male	2002	No treatment
Batra et al.[12]	15/Female	2004	Enucleation
Yamalik et al. [13]	51/Male	2007	Enucleation
Chew et al. [14]	30/Female	2008	No treatment
Imada et al. [15]	42/Female	2014	Marsupialization and enucleation

Some authors report that surgery is not the best treatment for dentigerous cysts. Two rare cases of bilateral dentigerous cysts were reported, in which the cysts underwent spontaneous regression without surgical intervention [7,10]. This case report emphasizes the importance of radiographic examinations of all unerupted teeth and using the orthopantomogram to arrive at an appropriate diagnosis and allow for better treatment for the patient.

## Conclusions

A bilateral dentigerous cyst is a very rare entity. A thorough examination should be performed to rule out any associated syndromes. Standard follow-up protocols for the periodic evaluation of the patient are warranted to detect any further clinical and pathological changes and prevent associated morbidity.
